# Lessons Learned From Early Undergraduate Exposure to the Medical School Curriculum: A Qualitative Analysis

**DOI:** 10.7759/cureus.59010

**Published:** 2024-04-25

**Authors:** Lauren Sugarman, Gary L Beck Dallaghan, Thomas Koonce

**Affiliations:** 1 Department of Medicine-Pediatrics, University of North Carolina School of Medicine, Chapel Hill, USA; 2 Department of Medical Education, University of Texas at Tyler School of Medicine, Tyler, USA; 3 Department of Family Medicine, University of North Carolina School of Medicine, Chapel Hill, USA

**Keywords:** clinical skills education, role model, medical school, pathway program, undergraduate education

## Abstract

Background: Students considering the health profession as a career rarely have an opportunity to explore medical school experiences. Pathway programs and “mini-medical school” programs exist but rarely involve integrating participants into the medical school experience. A novel for-credit undergraduate course was developed to embed students into a clinical skills course for medical students beginning in 2013. To better understand the impact of these experiences, this study explored former students’ perceptions and career trajectories.

Methods: Participants were contacted via email to participate in a virtual, semi-structured interview. Virtual interviews were recorded and transcribed verbatim. Three investigators analyzed 17 interview transcripts independently and developed a codebook. Investigators met to discuss common themes and outcomes.

Results: Participants received early education on patient interviewing and physical examination skills, health policy, and ultrasound. They noted their course experience was a productive way to gain insight into medical school and often cited it when applying for their chosen professional school. Although not a formal part of the course curriculum, many received guidance on the medical school application process, and some obtained letters of recommendation from physician facilitators. Participants emphasized the sense of belonging within the medical school community and affirmation of pursuing a health professional degree.

Conclusion: Participants found their experience to be meaningful and cited it as an influential factor in deciding to pursue a health professional degree. The course could be adopted by other institutions to enhance the variety of pre-health experiences for future medical students or health profession students.

## Introduction

As the US population grows and ages, the demand for health care is growing faster than ever before. By 2030, the US is estimated to have a deficit of over 139,000 physicians [1]. Some studies put this number as high as 200,000 [2]. Deficits in primary care [3], geriatric care [4], and rural medicine [5] are particularly stark. Based on these projections, medical schools have increased class sizes [6] or new schools have been created [7].

Numerous programs exist to foster student interest in a medical career. Though program formats may vary, early exposure to the medical field has been shown to increase interest in and pursuit of a medical career [8-12]. Most programs focus on high school students, but very few target undergraduate students. Fewer still provide the opportunity for undergraduates to be integrated into the actual medical school course.

Students considering a career in medicine may have many questions and reservations. Some of those questions center on the medical school experience and whether they can succeed within it. Though these questions and reservations can exist for all potential medical students, they are likely more of a limiting factor for those with insufficient access to pre-medical advising and support [13]. Therefore, at the University of North Carolina at Chapel Hill School of Medicine (UNC SOM), undergraduates have the opportunity to be directly integrated into the medical school curriculum through the Introduction to Clinical Medicine for Undergraduates (ICMU) course. The development of this course was informed by social learning theory [14]. This theory posits that through observation, modeling, and imitation, individuals learn. Additionally, it enhances learners’ sense of self-efficacy and motivation for learning [15].

The ICMU course provides undergraduate students exposure to and active participation in the medical curriculum’s clinical skills course. Based on the survey study that was conducted, undergraduate students reported benefits from the connections made with medical students who provided social and peer support as they contemplated a career in health care [16].

Findings from our previous study indicated that novel integration motivates the continued pursuit of education in the healthcare profession [16]. However, the survey offered only superficial details. This study sought to explore the contextual factors underlying the perceived impact of taking the ICMU course.

## Materials and methods

Qualitative approach

We approached this study using a social constructionism epistemology, which incorporates the researcher-researched relationship in the construction of meaning [17]. The interpretations offered were constructed through participant interviews, followed by discussions exploring deeper meaning by the research team.

This study was conducted using a qualitative narrative analysis approach [18]. Narrative analysis is used to explore individual’s personal stories to interpret the meaning of what they said. This method also allows for exploration of how the person expressed themselves, the language they used when describing a particular experience, and the thoughts and motivations they experienced.

Reflexivity

The research team consisted of a medical student and two faculty members. One faculty member is a physician and education leader who directs the ICMU course. The other is an education scholar with experience in qualitative research methods. The medical student had previous experience with the ICMU course. Because of the relationship of the course director with this study, the other two members of the team conducted the interviews and analyzed the majority of the data. This ensured any biases were minimized, but allowed the course director to provide context for some of the findings. As a group, regular discussions were held to ensure the meaning of the narratives was not misinterpreted due to personal experiences.

Sample

Individuals who completed ICMU were invited by email to participate in a semi-structured interview about their experience. Rosters of previous ICMU students were obtained from the course director. If a potential participant had already graduated from the University of North Carolina (UNC), their email was obtained from the UNC Alumni database. The ICMU students were then sent an email invitation explaining the purpose of the study and the contact to set up an interview. We contacted 360 students who had completed the course since its inception. Of those, only 25 responded to the invitation to be interviewed.

At the beginning of the interview, participants provided verbal consent to have the interview recorded for the purposes of generating a transcript. Video recordings were destroyed after transcripts were reviewed for accuracy to ensure the confidentiality of the participants. The UNC Institutional Review Board reviewed and approved this study (UNC IRB No.: 20-1879).

Data collection

A semi-structured interview script was prepared. The questions asked were intended to complement findings from a previous study [16] to provide more context to the survey questions. Open-ended questions were generated to further explore participants' experiences during the course and their plans after the course based on the previous study survey findings. The questions were discussed amongst the research team. A classmate of the lead author reviewed them for clarity. The interview protocol is available as a Supplementary Appendix.

The interviews were designed to run 30-60 minutes and were conducted via a Zoom video call (Zoom Video Communications, San Jose, CA). Interviews were conducted between September 2020 to May 2021. The interviews were recorded and transcribed verbatim for analysis. The final transcripts were de-identified for analysis. Video recordings of the interview were discarded after final transcripts were checked for accuracy and completeness. For instance, if the transcriptionist indicated something was not clear, that video would be reviewed to fill in the blanks; however, that step was unnecessary as the transcript review did not require rechecking the recordings.

Data analysis

The two members of the research team independently reviewed two transcripts to develop an initial codebook. Narrative analysis was used to explore how others make sense of their experiences and the world around them through the interpretation of their narratives [19]. The two researchers (LS, GLBD) independently coded 10 more transcripts. Upon completion, they met to resolve conflicts with their coding through discussion. Both felt they had achieved thematic sufficiency. To ensure no additional codes were identified, the coding framework was applied to five more transcripts by a third researcher (TK).

Upon completing the coding, the team consolidated the codes and constructed themes. This process was iterative and involved several conversations to determine the final themes.

## Results

A total of 25 interviews were conducted. Table 1 details the demographics of the entire sample. During the independent coding, no new codes were identified after 12 transcripts were completed. To verify this, the third researcher coded five more transcripts and did not identify new codes. There were seven major themes constructed from the analysis of the interview transcripts. Themes along with specific code words are listed in Table 2 and detailed below.

**Table 1:** Demographic data of interview participants

Participants (n = 25)	
Gender	
Women	16
Men	9
Age range	21-30 years
Career path	
Medical school	10
Applied to health professions program	11
Other career not in healthcare	4

**Table 2:** Codebook developed and used for data analysis of interview transcripts Codes were organized into overarching thematic categories.

Theme	Code words
Motivation to take the course	Healthcare career, volunteer, resume builder, medical school environment
Insights into medical school	Community, medical student learning, welcoming, diversity of experiences, insight into daily environment
Patient experience	Portrayal of patient experience, observed feedback, comfortable with self
Education	Physical exam, history taking, ultrasound skills, hands-on learning, discussion of sensitive topics
Improvements to the course	Increased exposure to the medical environment, more resources, question and answer (Q&A) sessions, capstone project
Belongingness	Inclusive, connection-building, medical students offered advice, shadowing experiences
Impact on the future	Reinforced prior decision, demonstrated qualities of medical school, visualized self as a medical student, little effect

Motivation to take the course

Students’ motivations varied depending on their background and desired career field. Many expressed interest in pursuing a career in health care, believing ICMU would be a good way to spend time with medical students and physicians. The course was often recommended by a friend or college advisor.

“I thought it would be a really neat way to gain some experience in the field…to be able to learn alongside medical students.” - Participant 1

“I had a pretty good idea I wanted to go pre-med before taking the class, and I knew this was a good way to both learn a little bit more about what med school's like and medicine in general, as well as something to put on my application that I thought would help me stand out.” - Participant 2

Participants were also motivated by the hands-on nature of the course. Many students wanted to see “a day in the life” of a medical student and get a sense of what a medical school’s environment could be like. Other students saw ICMU as a volunteer-like opportunity.

“I just really wanted to get in there and see what a day there was like for a medical student and…the rigor of classes, and how they learn how to treat.” - Participant 3

“I think I felt like I had a lot of experience with physicians, but not with medical students specifically… what my life was going to be like while in medical school… I already knew I wanted to be a doctor, and I knew what being a physician was like but being a medical student is pretty different.” - Participant 4

Insights into medical school

Because of the course structure, participants witnessed the learning process that medical students go through and how they are evaluated with live, simulated scenarios. Many students also commented on how participating in this course dispelled the myth of medical school being “cut-throat.” Specifically, participants noted that their ICMU experience promoted UNC’s School of Medicine as a positive, supportive community and made them more interested in attending UNC for medical school. ICMU students were also able to appreciate the diversity of medical students.

“UNC was on my list of medical schools that I was going to apply to and would love to get into. And it definitely helped me feel like that was a good decision. Seeing the interaction between the professor and the student, I was like…this is a place I would like to learn. This is a place I could thrive in.” - Participant 4

“I just liked having that opportunity to be in the med school and getting to talk to med students…getting a sense of what their classes and day-to-day is kind of like… it really just made me more excited about everything…it was like a personal development class for me.” - Participant 5

“It did represent the variation in medicine. How there are different kinds of people who come from different backgrounds, who have different stories of how they got to med school, that was interesting to learn about.” - Participant 6

Patient experience

ICMU students enjoyed their time being a part of the “patient experience.” They had the opportunity to portray patient cases and observe videos of medical students participating in patient simulations as part of their feedback. Some students commented how it was important to feel comfortable with their own body to be a successful standardized patient and ICMU student.

“Right now I'm…learning how to give a physical exam... I knew how…to make people more comfortable…what made me uncomfortable. I think being able to be the patient has helped me do the exam better.” - Participant 7

Education

While ICMU students were there to help medical students learn the art of history taking and physical examination skills, there was also a great deal of learning for them. Many ICMU students noted they learned interviewing and physical examination skills alongside medical students. ICMU students were also able to glean skills in ultrasound and other hands-on experiences. Some participants noted that witnessing patient-provider interactions during ICMU has influenced how they interact with their own patients today; similar to the “see one, do one, teach one” model that is implemented within clinical learning environments.

“The med students get to learn from this undergrad who's presenting them with some illness, but then also this undergrad is learning.” - Participant 5

“They did like tobacco cessation with me where they tried to coach somebody out of tobacco addiction. And then we also did some like sexual health encounters. I thought that was great exposure, talking about really intimate things that you may talk about with patients, and seeing how you navigate that in a professional way.” - Participant 3

Some participants who had taken the course when it was initially offered discussed workshops on topics related to careers in health care. Specific topics included writing a personal statement and healthcare policy.

Improvements to the course

As a means of continuously improving this course, participants were asked what enhancements could be made. Some suggested increasing the exposure to patient cases and other aspects of the medical school environment. Additional suggestions included some of the following ideas: having a capstone experience for students to interview standardized patients themselves, group activities with other ICMU students, and hosting physician/student panels.

“It would be nice if the students or the models got a handout or some kind of reading that was optional that they could do to learn more about the cardiac exam and what the students will be practicing on them.” - Participant 8

“Definitely more of the hands-on opportunities. I remember one class distinctly where we got to handle ultrasound technology and kind of test it on other people, and I thought that was really cool. If the ICMU students were offered similar experiences more often, I think that would have made the course a lot better and just would have set it over the edge.” - Participant 9

“I think it would have been kind of cool to…do a Standardized Patient Exam kind of as a capstone…” - Participant 10

Many former ICMU students said they would not change anything as the ICMU course was easy to fit into their schedule and was a low-stress commitment. The few students who took the course multiple times noted they enjoyed staying with the same group of medical students across semesters. Many participants mentioned they had recommended ICMU to their peers after completing the course.

Belongingness

A majority of participants noted they felt included among the medical students and staff. One student noted they were grateful to be a part of the entire three-hour session each week, rather than solely the physician examination portion. It allowed them “to do everything the medical students did.”

"You know how you get recorded when you do the Standardized Exams, we would watch over one or two people's and point out things that they could improve on. And so I got to participate in those discussions, which was kind of interesting. I really felt like I was a part of the class.” - Participant 10

“They [medical students] were all super nice and engaging with me. They kind of treated me like one of the medical students…I feel like I was engaged just as much as the medical students.” - Participant 3

Many ICMU students made connections with medical students and faculty during the course. Some used these connections to meet with medical students outside the course to discuss advice on applying to medical school. Additionally, many ICMU students noted they received advice and guidance during the course sessions. Others were able to obtain letters of recommendation from physicians that they used for the medical school application. Participants noted that working with the same group of medical students throughout the semester contributed to forming these connections.

“To be able to be a part of the medical school group in such an early part of my educational career, I thought was really great because then I had people that I could identify if I had a question… I could go to them and ask them that question…like am I doing the right thing with my academic planning, etcetera.” - Participant 1

“…my tutor was so sweet and acted like I was a medical student and tried to include me in teaching and stuff, which was really great. And he actually ended up writing me a letter of recommendation for medical school, and I think it really helped me get into med school.” - Participant 8

Some participants had a different experience, noting they did not form strong connections with the medical students or faculty. They indicated there was no form of mentorship or advice. Participants reported inappropriate comments from medical students and faculty facilitators creating a tense learning environment.

Impact on the future

The primary purpose of this study was to determine if ICMU students felt their experience in this course influenced their career decisions. Many noted that their ICMU experience solidified or reinforced their desire to go into medicine. A few participants had been undecided between different healthcare careers (doctor of medicine (MD) vs. physician assistant (PA), etc.) and ICMU helped them make their decision.

“Being able to picture myself in their shoes further motivated me to keep pushing towards my goal of becoming a physician.” - Participant 11

“It really solidified my desire to go to medical school, 'cause I just remember having so much fun in each PCC class and being so excited to learn about what the medical students were learning…” - Participant 8

The ICMU experience also helped some participants identify qualities that they would want in a health professional school, such as a team culture or supportive colleagues. ICMU allowed someone to visualize themselves as a medical student. Many participants noted that their ICMU experience fostered a desire to mentor other health professional students later in life.

On the contrary, some participants said their ICMU experience had little impact on their future other than having greater insight into what it was like to be a medical student. Participants who were interested in pursuing medical school during ICMU and later changed career plans noted that their ICMU experience was not one of the factors that swayed them away from a career as a physician.

## Discussion

The results of this study served two purposes. One was to contextualize the impact a novel undergraduate immersion course had on students considering a career in medicine or other health professions. The other was to identify what could be improved about the course. A previous study suggested this type of program encourages undergraduate students to pursue careers as healthcare professionals and facilitates their preparedness [16]. The present study suggests similar findings by contextualizing the experiences shared during participant interviews.

Participants suggested that ICMU helped jumpstart the development of their clinical skills, including history-taking and physical examination skills. While current health profession students receive ample training on these skills, early exposure does more than offer skill development. These experiences also contributed to the participants' sense of self-efficacy wherein they could see themselves in this role in the future [14,15].

In addition to influencing their self-efficacy, many of the participants also brought up how much the feeling of belongingness was important. The course served as a unique way to network with medical students and physicians. Participants were able to interact with medical students during the weekly sessions and sometimes outside of class time. Some participants were able to obtain letters of recommendation or shadowing opportunities from physicians. ICMU also familiarized undergraduate students with a portion of the medical student curriculum and the daily roles of medical students, similar to other programs that strive to connect students at various levels to physicians and other healthcare professionals [8,20,21].

A sense of belonging is defined as a fundamental psychological need. It is the degree to which an individual feels esteem (feeling included, valued, and respected), connectedness (being accepted, fitting in), and efficacy (feeling confident and capable) [22]. When individuals feel a sense of belonging, they experience increased confidence, professional satisfaction, and professionalism [23,24]. Many participants noted that they felt like they were a part of the medical school’s community. Cultivating a sense of belongingness has been shown to be an important factor for student motivation and learning identity formation [25]. Several students reported their ICMU experience made them want to attend UNC. In a previous study, 15/17 (88.2%) participants who matriculated into the UNC School of Medicine indicated that exposure to UNC SOM positively influenced their decision to attend the UNC School of Medicine [16].

One of the primary goals of this study was to determine the impact the ICMU course had on undergraduate students’ future career plans. Early exposure to the medical school curriculum and the healthcare field has been shown to foster and confirm interest in pursuing a medical career [8,11,26]. Almost all participants reported that ICMU affirmed their decision to pursue a career as a physician or helped them decide between different health professional degrees. All participants who were pre-health students prior to ICMU but then did not pursue medicine or another health professional degree reported that ICMU did not sway them from this path, but rather it was other factors. Thus, ICMU only acted as a positive or neutral experience when it came to impacting futures and helped many students become more certain of their future goals.

Participants suggested additional sessions for just ICMU students as a means of improving the course. Early cohorts of ICMU students had additional sessions on topics such as health policy and personal statement writing. Later classes of ICMU students expressed interest in these types of sessions to both deepen their knowledge base and develop deeper connections with other ICMU students, medical students, and physicians. Despite the desire for these additional connections, most participants reported they enjoyed the continuity of a single small group during regular course sessions.

The ICMU course has proven to serve several purposes in helping undergraduate students work toward their career goals and allowing UNC SOM to promote itself as an academic institution. The UNC SOM states in its mission that it “will recruit outstanding students and trainees from highly diverse backgrounds” [27]. Given that ICMU increases undergraduate students’ interest in UNC SOM, recruiting undergraduate students from underrepresented backgrounds for the ICMU course may advance UNC SOM’s commitment to diversity and inclusion. One study found that the curriculum exposure in mini-medical school programs helped mitigate the perceived barrier of lack of guidance/role models [28]. ICMU may function similarly as undergraduate students build connections with medical students and faculty.

Although the ICMU experience is greatly enjoyed by UNC undergraduate students, this study is limited in that it is from a single institution. Similar programs could be developed at medical schools, particularly if there is a nearby undergraduate institution. Not only do undergraduate students gain experience as medical students, but the medical schools gain additional standardized patients for sessions.

## Conclusions

This study further identified the impact a unique, immersive course experience has on undergraduate students considering a career in the health professions. The experience provides a benefit to undergraduate students, medical students, and faculty through hands-on activities and the sense of belonging participants feel. The course helped participants clarify what they wanted for a career due in part to this experience. Extending this type of course to other institutions is feasible and can provide benefits to all involved.

## Appendices

Supplementary appendix: Semi-structured interview protocol

Logistical Questions

- Could you state your age, and where you are in your schooling/professional career?

- When did you take ICMU (year/year in school)?

Pre-class

- What motivated you to sign up for ICMU?

- What were your career aspirations prior to starting the course?

- What were your expectations going into the course?

During the Class

- Describe your experience throughout the course

* What aspects did you like?

* What aspects you did not like?

* Were there aspects you felt were missing or wished there was more of?

. What was the level of responsibility and workload during the course? Do you wish it was different in any way?

. Can you describe your interactions with UNC SOM faculty and students?

* Did you feel included within the group?

* Did you feel like you could build connections with students and faculty?

- Have you sustained any of those connections or called upon them later on in life?

* Do you feel like this experience was meaningful? If so, what did it mean to you?

· Did you have any additional ICMU-related experiences outside of going to the weekly course (advising, meeting with students/faculty, workshops, etc.)?

Career Goals

- How did your ICMU experience influence your career goals?

* If you were pre-health, did you continue to be on this path?

* Did you change pre-health tracts (medical to nursing for example)?

* Did you switch out of or into a pre-health tract at least partially because of this course?

 - What role did ICMU play in making any future decisions related to a healthcare career?

* For undergrads, did you seek out other similar experiences?

* For graduating students/medical students, did it influence what medical schools you applied to or how you decided on a medical school?

View of UNC SOM

- How did the ICMU experience influence your view of UNC SOM?

Questions for Current/Past Medical Students

- If you did become a medical student, did this experience influence your desire to seek out mentorship roles for other undergraduate students?

- How has this experience influenced your clinical skills, such as taking a history, doing a physical exam, etc.?

- How much time was between you taking ICMU and applying for medical school?
